# Differences in structure of northern Australian hypolithic communities according to location, rock type, and gross morphology

**DOI:** 10.3934/microbiol.2018.3.469

**Published:** 2018-06-25

**Authors:** Susannah P. Guenther, Karen S. Gibb, Alea M. Rose, Mirjam Kaestli, Keith A. Christian

**Affiliations:** Research Institute for the Environment and Livelihoods, Charles Darwin University, Darwin, NT 0909, Australia

**Keywords:** extremophiles, hypolithic cyanobacteria, nematodes as vectors, microbial community, semi-arid zone, tropical, quartz

## Abstract

Hypolithic communities (under translucent rocks) were compared between a semi-arid site (Wave Hill) and a site with considerably higher rainfall (Lake Bennett) to test the hypothesis that the communities at the higher rainfall site would be more diverse. A total of 153 cyanobacteria operational taxonomic units (OTUs) were identified, and only 50 of those were found at both sites. Of these, only two were core OTUs, as defined as being present in ≥90% of samples, highlighting the extreme differences in the cyanobacterial communities at the two sites. At Wave Hill, we compared the composition of the cyanobacterial components under two different rock types (quartz and prehnite) to determine if the different minerals would result in different hypolithic communities, but no differences were found. Of the 42 core OTUs found at Wave Hill, 22 (52%) were shared between the two rock types. As hypothesised, the diversity of both cyanobacteria and eukaryotes in the hypolithic communities was significantly higher at Lake Bennett. Some hypolithic communities were thin and tightly adhered to the rock surface, but others were thicker and could be peeled off the rock in sheets. However, the two types were not significantly different in OTU composition. Metazoans, primarily nematodes, were ubiquitous, raising the possibility that nematodes may act as vectors to transport the components of hypolithic communities from rock to rock as a mechanism of colonization.

## 1. Introduction

Extreme environments, such as hyper-arid deserts, can present challenges for survival of most species [1]. These environments often include biological soil crusts and hypolithic organisms, dominated by cyanobacteria, that are increasingly recognized as performing important ecosystem functions [2]. These extremophiles play a significant role as primary producers, sources of atmospheric oxygen, nitrogen fixation, and soil stabilisers [3],[4]. For example, hypolithic microbial communities living under translucent rocks in the McMurdo Dry Valley in Antarctica are a significant source of biomass and productivity [5]. Studying these microbial communities yields important insights into the ways in which life can exist in extreme environments on Earth or elsewhere [6].

Species abundance in the hypolithic environment is dependent on the amount of water that is available from either fog or rainfall [7]–[9]. In addition to moisture, the composition of these communities is affected by the quality and quantity of radiation passing through the rock [10], the sodium content in the soil, and stochastic processes [11]. Translucent rocks such as quartz are common in many arid zones, and they provide physical stability, UV protection, photosynthetically active radiation, and increased availability of moisture that allows these communities to develop [1],[12],[13].

In addition to cyanobacteria, some hypolithic communities also include eukaryotes such as fungi, mosses [5] and lichens [13]. In some communities, there is a symbiotic relationship between lichens and cyanobacteria, with the cyanobacteria providing nitrogen fixation, phosphorus, and amino acids [14].

The objectives of this study were to compare hypolithic communities in northern Australia with respect to the composition of cyanobacteria and eukaryotes, type of translucent rocks, gross morphology of the hypolithic communities, and a comparison between a site in the semi-arid zone and a site with considerably higher rainfall. Based on previous studies, we hypothesized that the diversity of organisms composing the hypolithic communities would be greater at the higher rainfall site compared to the drier site. Qualitatively, the gross morphology (appearance) of the hypolithic communities in northern Australia ranges from a thin green smear under some rocks, to a thick, leathery structure on other rocks that can be peeled off intact. We compared these two morphologies to determine whether or not they were compositionally different. Recent studies of microbiota associations have resulted in the concept of a “core” community, based on near ubiquity (present in ≥90% of samples) [15],[16]. We compared the cyanobacteria core operational taxonomic units (OTUs) between the two sites and between two rock types at one site.

## 2. Materials and methods

### 2.1. Sites and sampling

Samples were taken from a semi-arid site ∼10 km south of Kalkarindji, Northern Territory Australia on Wave Hill Cattle Station (coordinates 17°34′10″S, 130°54′12″E) and from near Lake Bennett (coordinates 12°58′00″N and 131°00′50″S). The vegetation at the Wave Hill site is dominated by spinifex grass (*Triodia* sp.), with sparse shrubs and eucalyptus trees [6]. The Lake Bennett site is in an area of savanna woodlands in the wet-dry tropics of northern Australia, and is dominated by *Eucalyptus miniata* and *E. tetrodonta*, with an understory of *Acacia* shrubs, the palm *Livistonia humili*, and patches of *Pandanus spiralis*. Patches of rocky soil create areas that are relatively open, and we collected samples in one of these patches with few trees and sparse annual grasses. These sites are separated by a straight-line distance of 515 km, and the mean annual rainfall is 699.7 mm (range 241.3–1201.8 mm) at Wave Hill and 1521.7 mm (range 923.3–2065.7 mm) at Lake Bennett [17]. At Wave Hill, 20 samples were collected for cyanobacteria analysis (10 from quartz rocks and 10 from prehnite rocks) and 10 for eukaryotic analysis (quartz). At Lake Bennett, 10 samples were taken for cyanobacteria analysis and 10 samples were taken for eukaryotic extractions, all from quartz rocks because there was no prehnite at this site.

### 2.2. DNA extraction for cyanobacteria

Rocks were rinsed with high-pure water then scraped with a scalpel blade to sample both “thin” and “thick” microbial communities. These two types were categorized qualitatively, but they were distinct in that the thin samples tended to be tightly adhered to the rocks whereas the thick samples tended to peel-off the rock in leathery sheets. The samples were then placed into tubes from a DNeasy PowerBiofilm Kit [18] and stored at 4 °C until DNA was extracted the next day, following the manufacturer's instructions.

### 2.3. DNA extraction for eukaryotes

The proteins, polysaccharides, and other complex compounds in the cell walls of lichens can make it difficult to extract DNA by conventional methods, so we used a different technique to detect eukaryotes [19]. Samples for the eukaryotic extractions were collected as described above, and placed into 2.0 mL screw tubes with five sterilized 2 mm glass beads. Samples were snap frozen in liquid nitrogen and pulverized in a MoBio PowerLyser^®^ 24 at 3,200 rpm for 30 seconds and the DNA was then extracted with a KCl chloroform buffer [19]. Along with the samples, 4 negative controls (empty bead tubes) were included in the analysis.

### 2.4. Primers and amplicon description

The primers chosen for the cyanobacteria samples were CYA359F (5′-GGGGAATYTTCCGCAATGGG) and CYA809R (5′-GCTTCGGCACGGCTCGGGTCGATA) targeting the 16S rRNA genes of cyanobacteria [20],[21]. For the eukaryote samples, the following primer pair was used: 566F (5′-CAGCAGCAGCCGCGGTAATTCC), and 1200R (5′-CCCGTGTTGAGTCAAATTAAGC) targeting the region V4–V5 of the 18S rRNA gene [22].

The DNA MiSeq sequencing provider was the Australian Centre for Ecogenomics (ACE) at the University of Queensland. Sequences were processed by ACE using an in-house pipeline. The pipeline is on the forward reads only with the first 20 bases of sequences trimmed and poor quality sequences were removed using a sliding window of 4 bases with an average base quality above 15 using Trimmomatic. All reads were hard trimmed to 250 bases, and any with less than 250 bases excluded. Trimmed sequences were processed using the QIIME 1.9 open reference workflow [23] with default parameters (97% similarity). OTUs with an abundance of less than 0.05% of the total sequence abundance were removed. Taxonomy assignment was through BLAST against reference databases (Greengenes version 2013/05 for 16S). All cyanobacteria samples had more than 10,000 sequences and all eukaryote samples had more than 1,000 sequences. Samples were rarefied to the lowest common number of sequences, which was 11,384.

### 2.5. Statistical analysis

A square root transformation was applied to the OTU data to down-weight those OTUs that were highly abundant. Then, a Bray-Curtis similarity matrix was calculated using Primer-E v7 (Plymouth, UK) on the transformed data. A PERMANOVA analysis was conducted on the transformed data using site and community type (thin or thick) as fixed-factors to examine whether the microbial communities differed between these sample groups. SIMPER in Primer-E was used to determine which OTUs contributed most to the differences in site and community type. The Shannon diversity measures were calculated and compared for the different sample groups. This metric includes both OTU richness and evenness.

The core microbiota was defined as those OTUs that were present in at least 90% of samples within a group [15],[16]. The programming language R (version 3.3.3, using Vennerable) was used to create a Venn diagram to illustrate the relationships of core cyanobacteria between the sites and rock types.

## 3. Results

### 3.1. Composition

**Figure 1.**
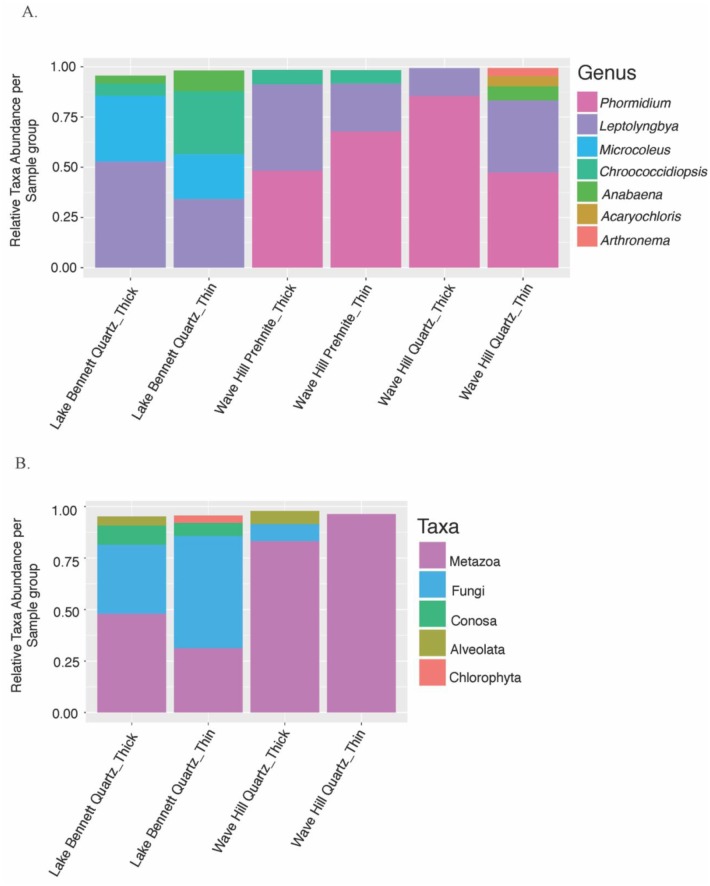
Taxonomic distribution of the components of hypolithic communities from two sites, Wave Hill and Lake Bennett. (A) Comparisons between sites, rock type, and gross morphology for cyanobacteria. (B) Comparisons between sites and gross morphology for eukaryotes.

After the filtering described above, 153 cyanobacteria OTUs were identified. Figure 1A shows a comparison between cyanobacteria at Lake Bennet and Wave Hill. The taxa bar plot supports the PERMANOVA result that there were clear differences in the composition of the communities between the two sites (Figure 1A; Table 1). Lake Bennett is the only site in this study where the genus *Microcoleus* was found. The genera *Phormidium*, *Acaryochloris*, and *Arthronema* were only found in the Wave Hill area, but the genus *Leptolyngbya* was found across both sites. In terms of gross morphology, thin and thick cyanobacterial communities were not significantly different in OTU composition, although the thin communities on quartz from Wave Hill had more OTUs than the thick samples (Table 1).

The examination of the Eukaryotic community also revealed differences in the communities at Lake Bennett and Wave Hill (Figure 1B). Both sites had Metazoa (Kingdom Animalia), Fungi (Kingdom), and Alveolata (a superphylum of protozoans). However, Lake Bennett also had Conosa (a subphylum of protozoans) and Chlorophyta (green algae).

**Table 1.** PERMANOVA results comparing the similarities of the cyanobacteria communities with respect to site, (Wave Hill and Lake Bennett), rock types (quartz and prehnite), and gross morphology of the community (thin and thick). Prehnite was only found at the Wave Hill site.

Organism	Factor	Pseudo-F (df)	P value	Unique Perms
Cyanobacteria	Site	21.7 (1)	0.001	998
	Rock type	1.3 (1)	0.156	999
	Gross morphology	0.8 (1)	0.662	996

Pseudo-F is the test statistic and ratio of variances in permutational multivariate analysis of variance (PERMANOVA). Unique perms is the number of permutations used in PERMANOVA to obtain the P-value.

### 3.2. Beta-diversity

**Figure 2.**
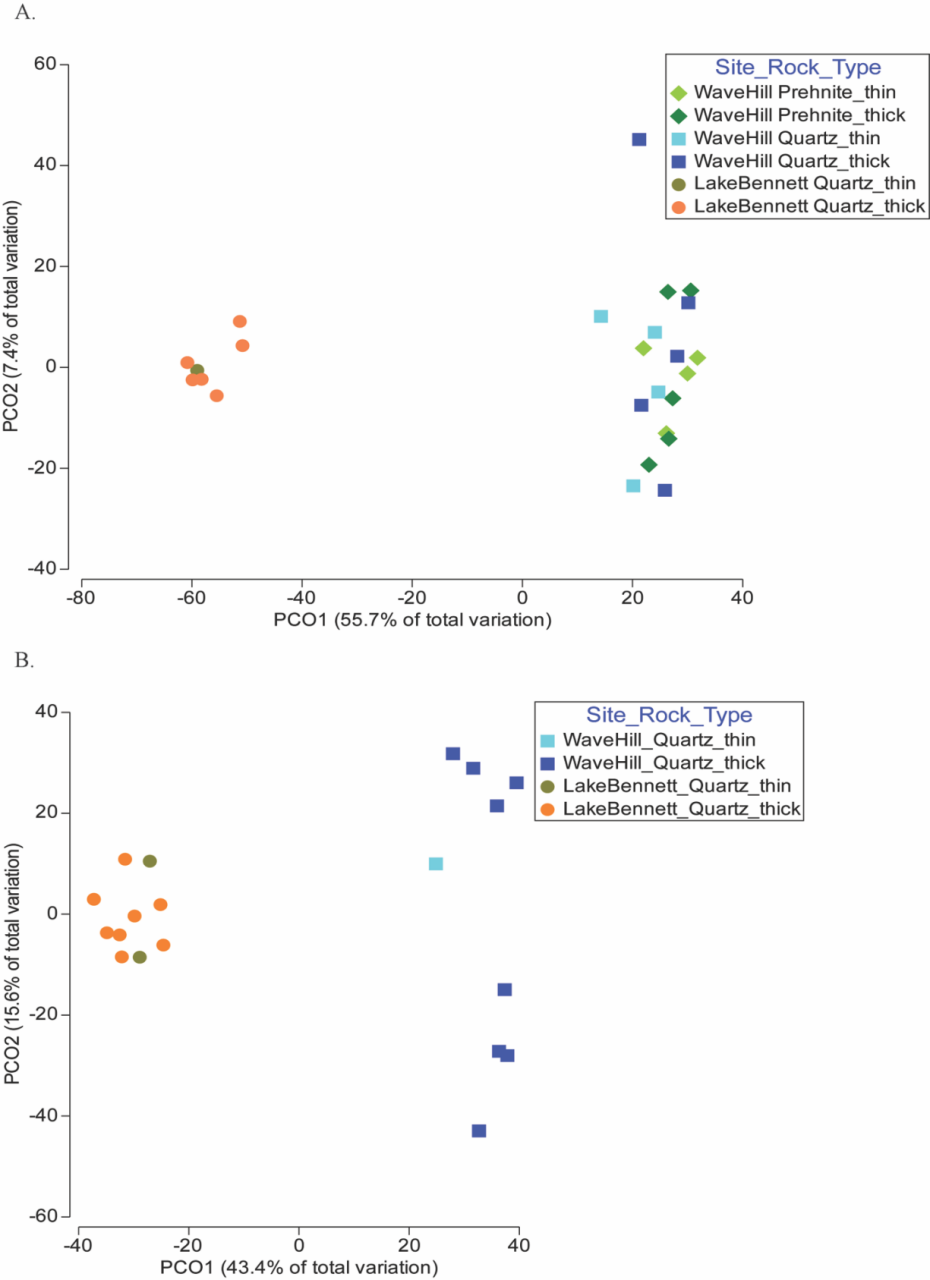
Principal Coordinate Analysis (PCO) of (A) cyanobacteria and (B) eukaryotes comparing sites (Wave Hill and Lake Bennett), rock types (quartz and prehnite), and gross morphology of the community (thin and thick). Prehnite was only found at the Wave Hill site.

A Principal Coordinate Analysis (PCO) in Figure 2A shows associations of the cyanobacteria OTUs on the rocks with respect to site, rock type and gross morphology. Site was the most important factor determining community composition whereas rock type and gross morphology did not significantly influence community composition (PERMANOVA; Table 1).

The PCO for the Eukaryotes (Figure 2B) also shows differences between the eukaryotic communities under quartz rocks from Wave Hill compared to those at Lake Bennett. Gross morphology had no influence on the composition. These patterns were confirmed by the results of the PERMANOVA analysis shown in Table 2.

Considering only the Fungi, the PERMANOVA results were similar to those for all Eukaryotes. That is, there was a significant difference between the two sites, but not between the thick and thin morphologies (Table 2). SIMPER analysis of the Fungi showed that, of the 20 fungal OTUs, the significant difference between Lake Bennett and Wave Hill fungal communities were mainly attributed to 9 fungal OTUs that were abundant on Lake Bennett quartz only. The Wave Hill fungal community was characterized by two groups, of which, one (*Hazardia* sp.) was unique to this site and contributed 52.5% to the similarity of the fungal population. Lake Bennett quartz, however, was dominated by five fungal OTUs.

### 3.3. Alpha-diversity

The mean Shannon diversity index of the cyanobacteria OTUs from Lake Bennett (2.93 ± 0.34 sd) was significantly higher than the diversity of cyanobacteria at Wave Hill (2.57 ± 0.44 sd) when compared by ANOVA (F_1,28_ = 5.16, P = 0.031).

The mean Shannon diversity index of the eukaryote OTUs from Lake Bennett (2.62 ± 0.47) was significantly higher than the diversity of eukaryotes at Wave Hill (2.06 ± 0.46) when compared by ANOVA (F_1,17_ = 6.68, P = 0.019).

**Table 2.** PERMANOVA results comparing the similarities of the eukaryote communities with respect to site and gross morphology of the microbial community on the underside of quartz rocks.

Organism	Factor	Pseudo-F (df)	P-value	Unique perms
Eukaryotes	Location	6.6 (1)	0.001	999
	Gross morphology	1.8 (1)	0.064	999
Fungi only	Location	6.3 (1)	0.001	998
	Gross morphology	1.2 (1)	0.250	997

Pseudo-F is the test statistic and ratio of variances in permutational multivariate analysis of variance (PERMANOVA). Unique perms is the number of permutations used in PERMANOVA to obtain the P-value.

### 3.4. Core OTUs

**Figure 3.**
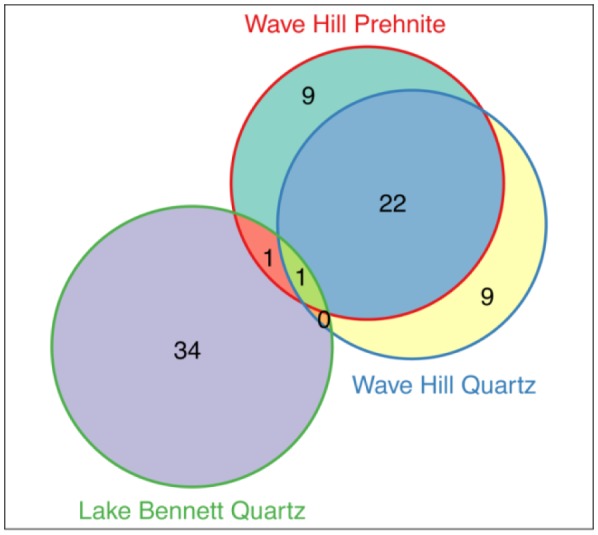
A Venn diagram showing the core cyanobacteria OTUs living under quartz and prehnite rocks at Wave Hill and under quartz rocks a Lake Bennett.

The numbers of core cyanobacteria OTUs from the two sites and the two rock types (at Wave Hill) are shown in Figure 3. Although 50 OTUs (of the 153 total) were shared between the two sites, only 2 of these were core OTUs. The two rock types at Wave Hill shared 22 core OTUs, corroborating the similarities between the communities on the two rock types shown by the PERMANOVA analysis (Table 1). Only 1 OTU was a core for all three groups of rocks (Figure 3).

## 4. Discussion

With respect to the objectives of this study, we found that: (1) Site had a significant effect on community composition and diversity with respect to both cyanobacteria and eukaryotes, with the higher rainfall site near Lake Bennett being more diverse. (2) There was no difference between the two rock types (quartz and prehnite) with respect to the community composition of cyanobacteria at Wave Hill. (3) Eukaryotes were found in many of the samples, but (4) there was no strong relationship between the gross morphology and the presence of eukaryotes.

Non-continuous patches of rock in the environment can mean that distance may be a barrier for species dispersal and distribution. Community composition in northern Australia is similar for rocks collected within an area of 4 m^2^, however, samples that were up to 100 m away were significantly different [24]. Thus, a distance of 515 km between samples would be expected to result in differences in community composition. However, environmental differences between the two sites are also likely to be important in determining the differences in community composition. The higher diversity at Lake Bennett (tropical savanna) compared to Wave Hill is consistent with previous studies which have shown a positive relationship between rainfall and abundance and diversity of hypolithic communities [7],[8],[11]. Interestingly, the only other hypolithic community previously reported from a tropical savanna (Thailand) was also distinctly different from the communities found in desert locations [25].

Transmittance levels of UVA, UVB and PAR through the rock can play a role in determining the composition, diversity and structure of microbial communities under rocks [10]. However, despite differences in the transmission of light through the two rock types [6], there were no significant differences between the hypolithic communities associated with quartz and prehnite samples from Wave Hill. This result is consistent with another study that included more than one rock type [26]. The variability among individual samples of a given rock type [6] may explain the lack of an effect of rock type. Thus, the effects of different combinations of factors such as rock thickness, discoloration, shape, position with respect to the soil, etc. may obscure the effects of differences in light transmission and quality due to rock type.

The community composition contains filamentous cyanobacteria, and these, along with lichens and the secretion of extracellular polymeric substance by the community [9], would contribute to the robust structure of the thick communities. Thus, the differences in morphology likely represent different stages of colony growth.

The cyanobacteria genus *Leptolyngbya* was found across all sites and in both the thick and thin community types. This genus is one of the most common cyanobacterial groups [27]. The genus *Phormidium* was common at Wave Hill, and it is known to naturally cluster with *Leptolyngbya* [27]. Interestingly, the genus *Chroococcidiopsis* is a relatively small component of the communities at the two Australian sites, whereas it dominates in nonpolar deserts on other continents [9].

The eukaryotes that combine with the cyanobacteria to form the hypolithic biofilm consist of Fungi and Chlorophyta, whereas the other eukaryotes, including the metazoans (such as nematodes) and protozoans are most likely exploiting this hypolithic biofilm for nutrients, shelter, or moisture. The hypolithic environment is rich in inorganic nutrients and bacteria which provide metazoans and protozoans with potential food substrates [28].

Fungi play an important ecosystem function as decomposers of organic material [29], and they can also form symbiotic relationships with cyanobacteria, in which the cyanobacteria supply energy and nitrogen to the fungus [30]. Fungi can use *Microcoleus* (found in samples from Lake Bennett) as a food source, and nematodes feed on fungi and cyanobacteria [31].

One of the questions that remains about hypolithic communities is: How does colonization of a rock occur? Although it has been suggested, based on the detection of hypoliths in soil, that selective microbes living in the soil can be recruited to form the hypolithic community [32]. However, as previously noted [24], the detection the hypolithic cyanobacterial DNA detected in the soil could represent either dormant [33] or relic [34] cyanobacteria rather than an active component of the soil microbiota. Microbes may disperse as bio-aerosols in desert dust [9], although as long as the dust is above-ground (as opposed to under a translucent rock), the hypolithic cyanobacteria would be exposed to much higher levels of solar radiation than they have evolved to exploit [6]. The long-term exposure to high levels of radiation in the open environment may have detrimental effects on the photosynthetic systems [35] (termed “photoinhibitory damage” [36]), whereas these photosynthetic systems are protected by the ultraviolet filtering provided by the host rock [10],[26]. Given the specialist nature of the microhabitat under translucent rocks [6], particularly the radiant environment, it is more likely that these specialist organisms must disperse from rock to rock [24]. Rock to rock dispersal of hypolithic cyanobacteria is consistent with the apparent evolutionarily isolated lineages at the global scale [9],[37], and significant differences among hypolithic communities at local and regional scales [24] (Figures 1 and 2 above) which indicate restrictions on dispersal rather than broad-scale dispersal mechanisms.

One proposed mechanism for rock to rock dispersal is by water runoff [38],[39]. However, the results in Figure 1B suggest the possibility of an alternative—that cyanobacteria and possibly fungi are transported by metazoans, such as nematodes. Microbes could be transported internally or externally by the nematode to new unexploited habitats (translucent rocks). When the bacteria pass through the gut of the nematode, the bacteria may be defecated alive, which would enable the bacteria to colonize a new area [40]. Although nematodes are known to transmit bacteria in soil [40], their role in establishing new hypolithic communities by moving them between rocks separated by areas inhospitable to hypolithic cyanobacteria (with respect to the radiative environment [6],[24],[36]) is not known.

## 5. Conclusions

A comparison of the two study sites showed that location is an important driver in hypolithic community composition and diversity, and the trends were consistent with the notion that hypolithic communities at locations with greater moisture availability are more diverse. There was no difference between the two rock types sampled at Wave Hill, and this is likely a result of the fact that many factors other than rock type can influence the hypolitic microhabitat and thus community composition and diversity.

In addition to a diverse cyanobacterial community [24], samples from northern Australia also support a range of eukaryotic organisms, including fungi, green algae (both of which would contribute to the physical structure of the hypolithic community) and protozoans and metazoans that inhabit the biological crust.

Although there is evidence that biological crusts, including hypolithic communities, perform important ecosystem functions such as soil stability, nitrogen fixing, carbon fixing, and moisture retention, these processes have not yet been studied at the ecosystem or landscape scale for hypolithic communities [2],[24]. Future work is needed to resolve the nature and magnitude of these ecosystem services. Furthermore, the mechanisms of colonization of hypolithic communities is unresolved, and the results of this study raise the possibility of transmission by nematodes.
